# Integrated Approaches of Arsenic Remediation from Wastewater: A Comprehensive Review of Microbial, Bio-Based, and Advanced Technologies

**DOI:** 10.3390/toxics13090768

**Published:** 2025-09-10

**Authors:** Aminur Rahman

**Affiliations:** Department of Biomedical Sciences, College of Clinical Pharmacy, King Faisal University, Al-Ahsa 31982, Saudi Arabia; marahman@kfu.edu.sa; Tel.: +966-(0)-547757460

**Keywords:** arsenic remediation, biochar, bio-based adsorbents, cost-effectiveness, fruit peels, microbial detoxification, nanotechnology, phytoremediation, sustainable remediation, wastewater treatment

## Abstract

Arsenic-containing wastewater and soil systems are a serious hazard to public health and the environment, particularly in areas where agriculture and drinking water depend on groundwater. Therefore, the removal of arsenic contamination from soil, water, and the environment is of great importance for human welfare. Most of the conventional methods are inefficient and have very high operational costs, especially for metals at low concentrations or in large solution volumes. This review delivers a comprehensive approach to arsenic remediation, including microbiological processes, phytoremediation, biochar technologies, bio-based adsorbents, and nanomaterial-assisted techniques. All of these methods are thoroughly examined in terms of removal competence, their mechanisms, environmental impact, cost-effectiveness, and scalability. Phytoremediation and microbial remediation techniques are self-regenerating and eco-friendly, whereas fruit-waste-derived materials and biochar provide abundant adsorbents, and are therefore low-cost. On the other hand, nanotechnology-based approaches show remarkable effectiveness but raise concerns regarding economic feasibility and environmental safety. Additionally, this review represents a comparative analysis and discusses synergistic and hybrid systems that combine multiple technologies for enhancing the remediation performance. Future research directions are emphasized along with challenges such as material stability, regeneration, and policy integration. This review aims to guide decision-makers, research scholars, and industry stakeholders toward affordable, sustainable, and high-performance arsenic remediation techniques for practical use.

## 1. Introduction

Arsenic (As), a naturally occurring metalloid, is well known for its toxicological relevance and harmful effects on human health and the natural environment. This metalloid is ranked as one of the top hazardous substances according to the U.S. Agency for Toxic Substances and Disease Registry [1]. Arsenic contamination in drinking water, agricultural soils, and natural ecosystems poses a significant public health and environmental threat worldwide, particularly in South Asian countries, including Vietnam, India, and Cambodia, which are disproportionately affected due to naturally occurring arsenic in sedimentary aquifers, as shown on the world map [2,3]. In addition, in Bangladesh, the arsenic-rich bedrock of the Brahmaputra river basin contaminates the groundwater as it is pumped up through hundreds of thousands of tube-wells and is consumed by millions of people [4].

**Figure 1:**
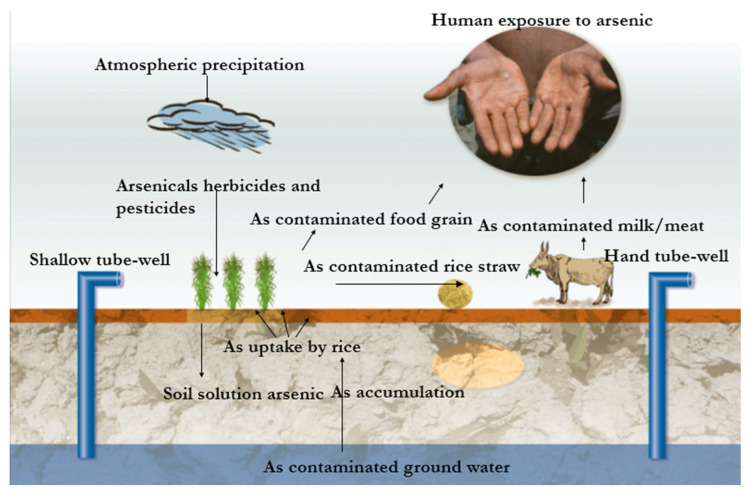
A diagram of arsenic contamination of the ecosystem, responsible for poisoning humans. Arsenic poisoning takes place directly through drinking contaminated water or consumption of contaminated foods, and indirectly via the meat–milk pathway.

Arsenic directly enters the environment through natural and anthropogenic pathways via three routes: (i) deposition of atmospheric particulates, (ii) disposal of arsenic-enriched sewage effluents, and (iii) by-products from arsenic mining processes and other processing industries. It is released into soils and groundwater through weathering, leaching from arsenic-rich bedrock, and geothermal activity such as volcanic emissions and hydrothermal vents [5,6]. Additionally, a considerable area of land is contaminated with arsenic originating from the use of sludge or municipal waste products. Human activities have markedly intensified arsenic release, especially mining, smelting, and the improper disposal of arsenic-rich wastes, along with car exhausts, residues from industries in glass production, pigments, semiconductors, and wood preservatives [7]. Combustion of arsenic-bearing coal also contributes to atmospheric deposition. Agricultural inputs are another major source of arsenic-based pesticides, herbicides, contaminated fertilizers, and irrigation with polluted groundwater, and they have left persistent residues in croplands [8]. Therefore, humans are considered the most highly exposed species to arsenic by directly and/or indirectly consuming arsenic-contaminated foods and water through the “plants–animal–human” pathway (Figure 1). Human exposure to arsenic occurs due to various factors, including anthropogenic activities like industries, atmospheric precipitation to the surface of the earth, using arsenic-containing herbicides and pesticides in agriculture fields, the irrigation of cultivated crops with arsenic-contaminated ground water, the uptake and accumulation of arsenic in plants, the consumption of arsenic-contaminated foods, feeding of metal-contaminated straw or leaves to cattle, the consumption of arsenic-contaminated milk and meat, and drinking metal-contaminated water pumped up using hand tube-wells.

**Figure 2:**
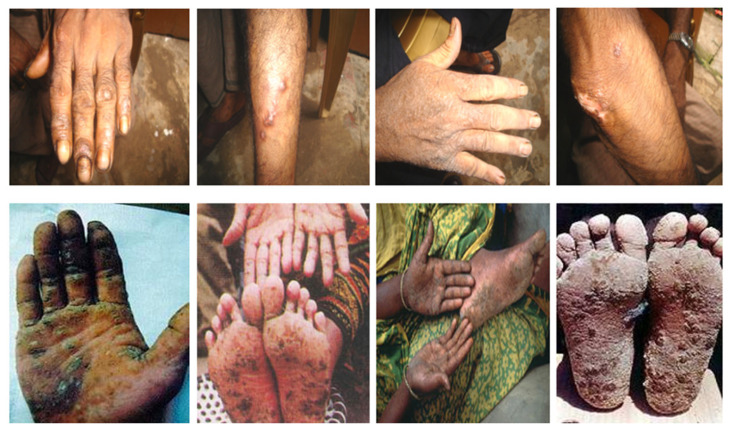
Effects of long-term exposure of humans to arsenic poisoning through drinking water and consumption of contaminated foods. These diseases are the direct evidence of human suffering resulting from arsenic contamination. Images adapted from Google search using the keyword “arsenic poisoning.” [Accessed on 1 July 2025: www.google.com/search?q=arsenic+poisoning].

Arsenics are mainly found in two oxidation states in aqueous environments, pentavalent arsenate [As(V)] and trivalent arsenite [As(III)]. Both of these forms are toxic to humans and the ecosystem, but As (III) is more toxic than As (V) [4]. Long-term exposure to arsenic is associated with carcinogenic, teratogenic, and mutagenic effects, causing skin lesions, diabetes, cardiovascular disorders, and a number of malignancies [9,10,11]. For example, Figure 2 represents the manifestation of arsenic poisoning, like keratosis and pigmentation. Spotty pigmentation (leucomelanosis) occurs in arsenicosis, diseases caused by long-term exposure to arsenics. Simple keratosis usually appears as bilateral thickening of the palms and soles, while in nodular keratosis, multiple raised keratotic lesions appear on the palms and soles. Moreover, skin lesions pose an important public health problem because advanced forms of keratosis are not only painful, but the consequent disfigurement can also lead to social isolation, particularly in villages in South East Asian countries [12].

Various anthropogenic activities, such as smelting, chemical industries, mining, the use of arsenic-based pesticides, and natural occurrences, continue to cause major environmental and health problems by releasing heavy metals into soil and water [10]. Therefore, it is urgent to remove arsenic from the water and soil systems.

Several techniques for removing arsenic from wastewater and soil systems have been studied. Among such methods, bioaccumulation, microbial degradation, coagulation, ion exchange, chemical precipitation, evaporation, phytoremediation, membrane filtration, reverse osmosis, electro-floatation, solvent extraction, electrocoagulation, electrodeposition, and electro-dialysis have been studied for arsenic removal from aqueous environments [13,14,15,16,17,18]. These traditional methods are effective under controlled conditions. However, these methods have some difficulties and disadvantages due to the production of secondary wastes, incomplete removal of trace-level heavy metals, a large quantity of slug formation, consumption of large amounts of chemical reagents and energy, generation of toxic byproducts, requiring sophisticated infrastructure and skilled operation that requires further treatment, and high operational costs [19,20,21]. Therefore, there is an urgent need to develop and implement environmentally friendly, cost-effective, and socially acceptable alternatives that can be adjusted to different water matrices and scales.

Due to the limitations of conventional methods, many eco-friendly and sustainable techniques have been developed in recent years. These include microbial remediation, which uses bacteria that are arsenic-oxidizing or -reducing bacteria for biotransformation and immobilization [4,12,22]. Bio-based adsorbents, derived from agricultural waste such as cellulose fibers, fruit peels, which are abundant, cost-effective, and biodegradable [23,24]. Phytoremediation, where plants such as *Pteris vittata*, *Arabidopsis thaliana*, and *Tobacco* are used to uptake and stabilize arsenic from soil and water [14,25,26]. Biochar and engineered biochar made from biomass, providing high surface area and adjustable adsorption qualities adsorption properties [27]. Nanotechnology-based solutions that use iron-based materials such as iron-based layered double hydroxides (LDHs), iron-based nanoparticles, zero-valent iron (ZVI), iron-doped polymer/biomass materials, iron-doped activated carbon, iron-doped inorganic minerals, and iron-containing combined metal oxides, graphene oxide, and carbon nanotubes for effective arsenic adsorption [28,29]. Individually, these techniques are effective and show promise in arsenic remediation. However, when these techniques are integrated into hybrid systems, they exhibit much more promise. Individual restrictions can be overcome, for instance, the synergetic effect of microbes and fruit peel cellulose, plant-based systems strengthened with nanocomposites, or microbial consortia supported by biochar. This combination increases the effectiveness of the cleanup of heavy metals [30].

There are a large number of studies on arsenic remediation using individual technologies, but there is a lack of comprehensive reviews that integrate all significant green and advanced technologies and critically assess their performance, limitations, scalability, and cost-effectiveness. Most of the existing reviews focus either on physicochemical processes or specific technologies, without making comparative conclusions across biological, bio-based, and nanomaterial platforms [31,32]. This review fills that gap by providing a comprehensive assessment of arsenic remediation technologies divided into three major categories: (i) microbial techniques, including bioaccumulation, reduction, and oxidation, (ii) bio-based and natural adsorbents, including plants, cellulose, fruit peels, and charcoal, and (iii) advanced technologies, including hybrid nanocomposites and nanomaterials. Each technique is discussed in terms of its mechanism, benefits, drawbacks, environmental effects, and viability with a focus on comparative efficiency, economic feasibility, environmental sustainability, field applicability, and scalability. The principal aims of this review are to present a current, multidisciplinary summary of emerging and established arsenic cleanup techniques utilizing natural, biological, and advanced technologies, as well as to critically evaluate these methods based on knowledge gaps, technical performance, limitations, cost, and sustainability. Finally, this article will suggest possible integrated and hybrid development techniques that are useful for implementing arsenic cleanup in rural and resource-constrained areas. This review is proposed to be a valuable reference for wastewater engineers, environmental scientists, public health professionals, and policymakers involved in designing arsenic-safe water treatment systems around the world.

## 2. Review Methods

Different electronic databases such as ScienceDirect, PubMed, PubMed Central, Web of Science, and Google Scholar were searched for the available literature on arsenic bioremediation from 2010 up to 2025. However, few studies conducted before 2010 were included in this review, as we found them important for the explanation of context. Literature searches were conducted using keywords: “Arsenic remediation”, “microbial arsenic resistance”, “microbial methylation of arsenic”, “biochar”, “bio-based adsorbents”, “biological volatilization of arsenic”, “cost-effectiveness”, “fruit peels”, “microbial detoxification”, “nanotechnology”, “phytoremediation”, “phytoremediation mechanisms”, “hyperaccumulator plants”, “sustainable remediation”, and “wastewater treatment”. All articles from the search were independently analyzed by title, abstract, and selected full text. Full-text journal articles published in English were included. Studies were excluded if the full-text article was unavailable or if the articles were in languages other than English. Letters to the editor, conference abstracts, and book chapters were excluded.

## 3. Microbial Remediation of Arsenic

Microbial remediation is a notable feature for its environmentally friendly nature and ability to remove both As(III) and As(V). Certain arsenic-oxidizing and arsenate-reducing bacteria can transform arsenic into less toxic or more easily removable forms. Microbial remediation provides a cost-effective, sustainable, and eco-friendly solution for arsenic detoxification in contaminated water and soil systems.

**Figure 3:**
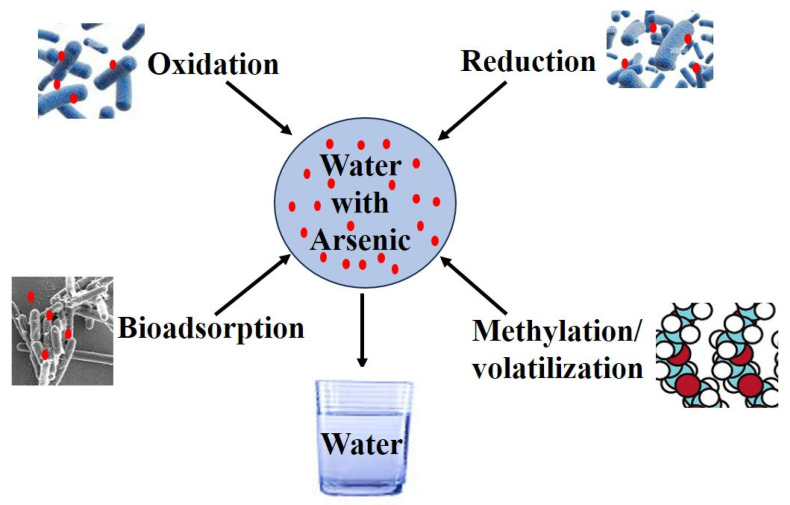
A schematic illustration of the main microbial arsenic transformation pathways (oxidation, reduction, bioadsorption, and methylation/volatilization).

Naturally occurring and genetically engineered microorganisms have shown the ability to transform or metabolize various arsenic species using several mechanisms, including biosorption, reduction, oxidation, and methylation [33], as shown in Figure 3. These reaction systems can detoxify arsenic or convert it into less toxic forms, thereby facilitating its removal from aquatic environments [34]. Microbial remediation is effective in aerobic and anaerobic conditions. For example, arsenite-oxidizing bacteria can convert the more toxic form of arsenic into the less toxic form of arsenic, which is As(V), and can be effectively removed through adsorption onto microbial biomass [35]. This biochemical pathway involves such mechanism in which the arsenite oxidase enzyme facilitates the conversion of As(III) to As(V) in aerobic conditions [36]. This process is primarily carried out by heterotrophic and chemoautotrophic bacteria, including species of *Agrobacterium*, *Acinetobacter*, *Alcaligenes*, *Pseudomonas*, and *Thermus* [36,37,38]. These bacteria express the *aio* gene cluster, which encodes arsenite oxidase, the key enzyme catalyzing the oxidation of As(III). For example, *Pseudomonas putida* and *Agrobacterium tumefaciens* have been extensively studied for their efficient As(III) oxidation capabilities [38].

On the other hand, arsenate-reducing bacteria may immobilize arsenic by precipitating it with other minerals. This process is mediated by enzymes such as the cytoplasmic ArsC or the respiratory arsenate reductase ArrAB, under anaerobic conditions, to reduce As(V) to As(III) [4]. This process is primarily carried out by arsenate-respiring bacteria (AsRB), such as *Shewanella*, *Geobacter*, *Lysinibacillus, Desulfotomaculum*, and *Chrysiogenes arsenatis* [4,39,40].

Some microorganisms are capable of methylating arsenic into volatile organic species commonly observed in fungi and some bacterial genera like *Aspergillus nidulans*, *Clostridium*, *Aspergillus* sp., *Paraclostridium* sp., *Cytophagaceae* sp., *Escherichia coli*, and *Methanobacterium* [34,41,42]. The arsenic methylation and subsequent volatilization are driven by arsenic methyltransferases (ArsM), which convert inorganic arsenic into methylated compounds such as monomethylarsonic acid (MMA), dimethylarsinic acid (DMA), and volatile arsines [41,43]. A study shows that the abundance of arsM genes in anaerobic granular sludge increased by 34.62–129.05% after the 100-day incubation, and was strongly correlated to arsenic volatilization [44]. Another study introduced the arsM gene from Rhodopseudomonas palustris CGA009 to construct an *E. coli* AW3110 co-expressing arsB/acr3 and arsM, which exhibited higher As(III) resistance [45]. However, this process may pose environmental risks due to the partial toxicity of intermediate compounds.

An important microbial-based arsenic remediation involves the formation of scorodite (FeAsO_4_·2H_2_O). Scorodite is a common arsenic-bearing iron mineral that could immobilize or store arsenic in a bound state [46]. It is a highly stable compound for arsenic immobilization, traditionally synthesized under high arsenic concentrations and extreme conditions, such as elevated temperatures and pressures. This process is mediated by iron-oxidizing bacteria, which oxidize ferrous iron (Fe^2+^) to ferric iron (Fe^3+^) under acidic conditions [47], that could immobilize or store As in a bound state [48]. The ferric iron then reacts with arsenate (As^5+^) to precipitate scorodite, effectively immobilizing arsenic in a crystalline form. A study demonstrated that the efficiency of arsenite oxidation was over 99%, with a maximum specific oxidation rate of 280 mg As(III) using scorodite [49]. According to Tanaka et al. [50], the process of removing arsenic through the formation of biogenic scorodite involves two steps: first, microbial oxidation produces amorphous precursors that contain SO_4_^2−^, which are then dissolved and recrystallized into stable crystalline scorodite that immobilizes arsenic. The process of arsenic remediation has been extensively studied and optimized for various conditions, demonstrating its efficacy in both laboratory and field applications.

Biosorption and bioaccumulation are the most widely studied mechanisms in microbial arsenic removal. Microorganisms can remove arsenic through bioaccumulation and biosorption, involving the uptake and binding of arsenic to their internal compartments or cell surfaces [51]. These arsenic-transforming metabolic actions are controlled by the ars operon, a genetic element widely found in arsenic-resistant bacteria and archaea [52]. Certain bacteria and fungi have cell walls rich in functional groups such as hydroxyl, carboxyl, and phosphate moieties that facilitate the binding of arsenic ions [53]. *Rhizopus arrhizus*, *Acinetobacter* sp., *Bacillus subtilis*, *Lysinibacillus* sp., *Talaromyces* sp., and *Saccharomyces cerevisiae* have shown high arsenic uptake capacities [4,36,54,55]. Immobilized biomass or dead cells are often employed to enhance adsorption capacity and reduce the risks associated with live microorganisms [56].

**Figure 4:**
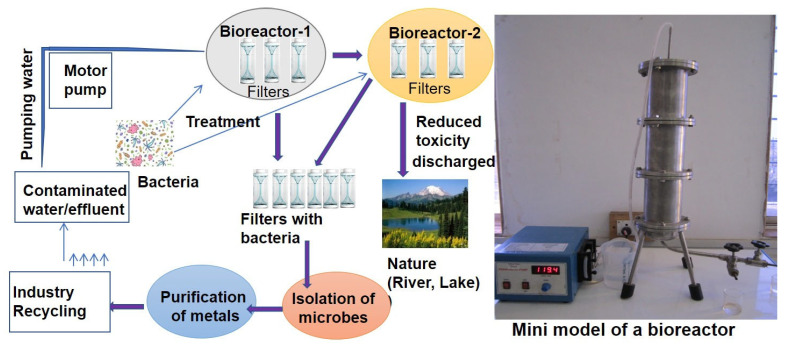
A schematic diagram of the microbial remediation of arsenic disposed of as either effluents or solid wastes from the industries or by other anthropogenic activities. The figure is adapted from the author’s previous publication [12], with slight modifications.

Recent advances include genetically engineered bacteria overexpressing ars operons or aio genes with enhanced arsenic transformation or enhanced arsenic sequestration capabilities [57]. Furthermore, genetic engineering is opening new approaches to improving microbial transformation and arsenic tolerance capacity. For example, transgenic strains of *E. coli* and *Pseudomonas* sp. expressing arsenic oxidase genes have demonstrated significantly improved remediation potential in laboratory settings [58,59]. Simultaneously, bioreactor systems that utilize immobilized arsenic-transforming bacteria show great promise for continuous arsenic removal from wastewater [60]. These systems are optimized for environmental parameters such as pH, temperature, and redox potential, allowing for efficient, continuous arsenic removal [61]. These systems are predominantly promising in applications such as constructed wetlands. A microbial bioremediation work proposal is depicted in Figure 4, where arsenic-contaminated wastewater is collected by a motor pump and treated several times with arsenic-accumulating bacteria. Bacteria accumulate arsenic, and thus the wastewater becomes decontaminated then the arsenic-free water is released into nature.

Microbial remediation is a promising, eco-friendly, energy-efficient process, which is safe for the environment, and appropriate for in situ applications that leverage natural biogeochemical processes [36]. Table 1 summarizes the important microorganisms involved in arsenic remediation, highlighting their type, mechanism of action, arsenic species targeted, key enzymes, optimal conditions, and reported efficiencies. The data show that microbial remediation generally achieves moderate efficiency, often lower compared to other bio-based techniques such as adsorption on cellulose and biochar. Nevertheless, microbial remediation of arsenic faces several limitations. These include sensitivity to environmental fluctuations, slow reaction rates under certain conditions, and competition with native microbial populations [62]. Microbial activity is highly sensitive to environmental factors such as temperature, pH, and redox potential [63]. Under anoxic conditions, there is a risk of remobilizing As(III), which poses additional challenges. Additionally, complete detoxification often requires integration with other treatment methods such as adsorption or filtration to remove transformed arsenic species from the water phase [64]. However, microbial treatments typically require controlled environmental conditions (pH, temperature, nutrients) and longer treatment times, which may limit large-scale distribution without proper bioreactor systems.

## 4. Cellulose and Fruit-Peel-Based Adsorbents for Arsenic Remediation

The biomass-derived materials, especially from agricultural waste and fruit peels, are excellent cellulose-rich bioadsorbents. They are sustainable, low-cost, environment friendly, and effective adsorbents for arsenic removal [86]. These resources are perfect for environmentally friendly water treatment solutions since they are abundant, renewable, biodegradable, have potential for regeneration, and are typically non-toxic [87]. These materials contain cellulose, hemicellulose, lignin, and pectin. Their surface provides active functional groups such as carboxyl, hydroxyl, and carbonyl moieties [88]. The functional groups play a significant role in binding arsenic ions through surface complexation, hydrogen bonding, facilitating ion exchange, or electrostatic interactions [89]. However, such adsorbent materials often require minimal processing and make them suitable for use, offering high potential for implementation in low-resource settings [90].

Among the most studied cellulose-based adsorbents derived from agricultural byproducts have been used for treating industrial effluents are wheat husk [91], rice husk [92,93], corn husk [92], sawdust [94], peanut husk [95], apple peel [96], banana peel [97,98], orange peel [23,99], lemon peel [100], watermelon rind [101], potato peel [98], sunflower biomass [102], sugarcane bagasse [103], coconut shell [104], waste tea leaves [105], marine algal biomass [106], water hyacinth [107], moringa seeds [108], shrimp shells [20,21], charcoal [109], etc. These materials may be used raw or chemically modified to enhance their adsorption properties. The chemical treatments increase surface area, porosity, and the number of active binding sites and introduce more reactive functional groups [110]. The adsorbents demonstrated high adsorption competence due to their porous structure and the presence of natural polysaccharides like cellulose, pectin, and lignin [88]. For instance, arsenic was effectively removed with the banana peel at an adsorption percentage of ~37% has been demonstrated to facilitate this due to their abundance of carboxylic groups [111]. Arsenic adsorption reached 98.50% at neutral pH using shrimp-based chitosan, indicating highly efficient removal under environmentally relevant conditions [20]. In addition, the affinity of cellulose-based adsorbents for As(V) and As(III) ions can be greatly increased by treatment with iron salts (e.g., FeCl_3_, FeSO_4_). Iron oxide and its composite adsorbents are widely used for removing arsenic. An adsorbent of iron- and zirconium oxide nanoneedle-impregnated cellulose nanofibers (Fe-Zr-NN-CNF) demonstrates a removal efficiency of 98% across a broad pH range of 2 to 9 [112]. The applications of cellulose-based (nano) composites as adsorbents have great potential in removing arsenic and other heavy metal ions, as well as organic pollutants [86]. Iron-loaded cellulose composites have demonstrated high removal efficiency of As(III) (99.6 mg/g) and As(V) (33.2 mg/g) at pH 7.0, due to the strong binding affinity between As(V) and ferric hydroxides formed on the cellulose surface [113].

The chemical modification of both cellulose and fruit peel materials can be performed through alkaline treatment, acid hydrolysis, impregnation, or esterification with metal oxides or nanoparticles [114]. However, it is important to consider that such chemical modifications may introduce potential expenses, chemicals, and increased waste, which must be considered against the enhanced performance.

Overall, cellulose and fruit-peel-derived adsorbents represent a promising class of low-cost materials for arsenic removal from wastewater [115]. Biomass-based adsorbents face certain limitations despite their potential. Their unmodified forms may often have low adsorption capacities and are vulnerable to microbial contamination and degradation [116]. Furthermore, their performance is highly pH-dependent, with neutral to slightly acidic pH ranges (pH 5–7) typically exhibiting the best arsenic removal [117]. The regeneration and reuse of natural biosorbents can also be challenging, although recent studies have indicated promising regeneration efficiency utilizing mild desorption agents such as NaOH or citric acid [118,119]. However, investigations are ongoing to overcome these limitations by developing composite materials combining fruit peels with nanoparticles, polymers, or biochar to enhance stability and capacity [119]. Continued investigation is needed to standardize their preparation techniques, test their long-term performance in actual wastewater, and determine their scalability and environmental impact under field conditions, especially in arsenic-endemic regions of South Asia, Africa, and South America [120]. Table 2 represents a comparative analysis of agro-based adsorbents, highlighting their efficiency, adsorption capacities, advantages, limitations, and operational parameters for arsenic removal from aqueous solutions.

## 5. Plant-Based (Phytoremediation) Techniques of Arsenic Remediation

**Figure 5:**
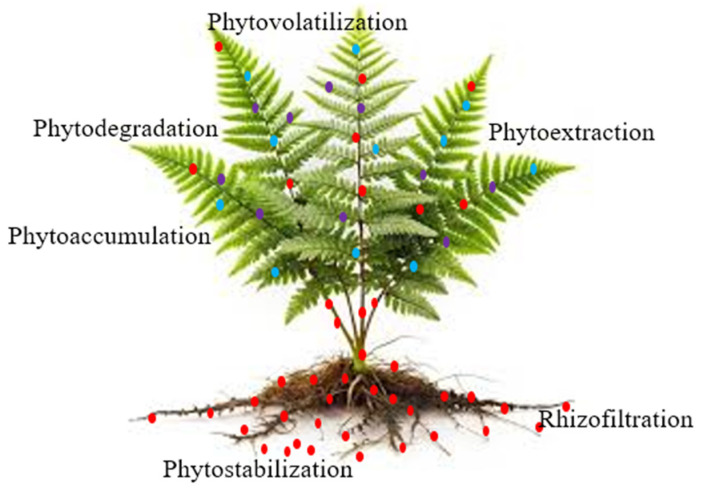
A schematic view of arsenic-accumulating plants to remediate soil and water contamination. These plants can accumulate heavy metals in their roots and translocate them to their shoots, which can be extracted, degraded, or volatilized. Red, blue, purple dots denote total arsenic, arsenite [As(III)], arsenate [As(V)], respectively.

Plant-based phytoremediation is a green and cost-effective approach that uses plants and their associated rhizospheric microbes to remove, stabilize, or transform contaminants, including arsenic, from wastewater, wetlands, and polluted soil systems [144,145]. This eco-friendly remediation technique takes advantage of the natural ability of plants to absorb arsenic and either store it in their tissues or convert it into less toxic forms. The phytoremediation method functions through various processes to eliminate arsenic from the environment. Several phytoremediation techniques, including rhizodegradation, phytodegradation, phytostabilization, phytovolatilization, phytoextraction, and phytofiltration (Figure 5), showed a number of benefits in terms of cost-effectiveness, user-friendliness, and environmental compatibility in removing arsenic from contaminated water and soil systems [144]. In phytoextraction, plants take up arsenic from the water or soil systems and store it in their above-ground parts, such as leaves and stems [146]. For example, *Pteris vittata* can take up 126-fold arsenic in the shoots versus soil, without the need for chelating agents or other soil amendments. In rhizofiltration, the plant adsorbs arsenic onto the surfaces of plant roots. Favas et al. have demonstrated that many plant species are able to remediate arsenic by this process. For example, the highest concentration of arsenic was found in *Callitriche brutia* (523 mg/kg DW), *Callitriche lusitanica* (2346 mg/kg DW), *A. caroliniana* (397 mg/kg DW), *L. minor* (430 mg/kg DW), *R. trichophyllus* (354 mg/kg DW), *Fontinalis antipyretica* (346 mg/kg DW), and *Callitriche stagnalis* (354 mg/kg DW) [147]. In phytostabilization, the plants limit arsenic mobilization in the soil around the roots. In plant root vacuoles, arsenic is bound through to ferric sulfate, forming a trivalent complex of As-tris-thiolate in the rhizosphere [148]. In phytovolatilization, the plants alter arsenic into a gaseous form to release it into the atmosphere. Phytovolatilization typically transforms inorganic arsenic into volatile methylated arsines such as monomethylarsine and dimethylarsine [149]. This step involves a series of steps for arsenic removal. First, plants take up arsenic from the soil, transform the less volatile compounds into more volatile forms, and then release the pollutants to the atmosphere via the volatilization process [146]. For example, by volatilization, *S. maltophilia* is resistant up to 165.00 mg/L of As(III), while *Agrobacterium* up to 80.00 mg/L of As(III) [150]. Phytoextraction and rhizofiltration are the most efficient arsenic-removing phytoremediation techniques from aqueous environments [146].

Several plant species have shown outstanding capabilities of absorbing arsenic. Among them, *Pteris vittata* is a well-known hyperaccumulator fern that can absorb large amounts of As(V) and transfer it to its leaves or branches [151]. Other potential plant species include *Arabidopsis thaliana* [25], *Brassica juncea* (Indian mustard) [152], *Helianthus annuus* (sunflower) [153], and *Typha latifolia* (cattail) [154], which are being investigated for their phytoremediation competence in both water and soil systems. These species are chosen for their high biomass production, rapid growth rates, and environmental adaptation to different conditions, making them appropriate candidates for large-scale applications [146,150].

The mechanisms of arsenic uptake and detoxification in plants are involved in both biochemical and physiological processes. As(V) is absorbed by phosphate transporters due to its chemical resemblance to phosphate ions, and subsequently As(V) is reduced to As(III) by arsenate reductases within the plant [155]. The more hazardous As(III) is then either released from the root system by efflux transporters or sequestered into vacuoles by forming complexes with thiol-rich peptides such as phytochelatins. These mechanisms help to reduce toxicity and enhance the plant’s ability to tolerate and accumulate arsenic [14,156].

Despite its advantages, phytoremediation still suffers from several shortcomings, including slow growth of plants, being time-consuming and sensitive towards heavy metals, and might not be the ideal option for urgent remediation needs [157]. For example, the strongest accumulator, *Pteris vittata*, may take several years to reduce the arsenic contamination from soil, depending on the initial contamination load and environmental conditions. Recent studies indicate that *Pteris vittata* can decrease arsenic after three years [158], 51% reduction by one year [159], and accumulation of 43.5 g of As per 154 m^2^ in 8 years [160]. The phytoremediation process may be affected by climatic and edaphic factors, and may require post-treatment of leachate or the disposal of biomass. Furthermore, improper disposal of arsenic-containing plant biomass may decompose or enter the food chain, causing secondary pollution. The outcomes of phytoremediation are significantly influenced by environmental factors like soil chemistry, climate, and seasonal variations [161]. In order to overcome these limitations, several researchers aim to use biostimulants, microbial-assisted phytoremediation (rhizoremediation), and transgenic plants to increase stress tolerance and metal uptake efficiency [162,163].

In summary, phytoremediation is a viable and long-term approach to arsenic cleanup, especially in environments with limited resources. The success of phytoremediation depends on the selection of appropriate hyperaccumulator plant species, thorough in-depth knowledge of absorption mechanisms, and careful post-harvest management of biomass [161,164]. Integrating phytoremediation with microbial, chemical, or nanotechnology-based methods could further enhance its effectiveness and suitability across a variety of environmental contexts [165]. Table 3 summarizes the commonly used plant species for arsenic phytoremediation, detailing their type, uptake capacities, tolerance levels, mechanisms, and advances of arsenic accumulation.

## 6. Biochar and Modified Biochar for Arsenic Remediation

Biochar, a porous carbonaceous substance made by pyrolyzing biomass in an oxygen-limited environment, has emerged as a promising material for the remediation of arsenic-contaminated water and soil systems [190]. It has a renewable nature, affordability, and adaptability in eliminating both inorganic arsenic species, As(III) and As(V), by the combination of chemical interaction and physical adsorption mechanisms. Biochars resulting from agricultural waste or biomass demonstrate excellent arsenic adsorption capacity, especially when chemically modified or functionalized with metals like iron or manganese. Owing to its large specific surface area, extensive porosity, and diverse surface functional groups (e.g., hydroxyl, carboxyl, phenolic), biochar demonstrates a strong attraction for pollutants, especially when customized or modified for targeted applications [191].

The adsorption effectiveness of biochar is significantly influenced by the pyrolysis temperature, post-treatment modifications, and feedstock composition [192]. For example, the physicochemical properties of biochars derived from agricultural waste like rice husks, corn stalks, coconut shells, and sugarcane bagasse vary in their physicochemical characteristics, which, in turn, influences their arsenic removal efficiency [193]. Biochars produced at lower pyrolysis temperatures (between 300 and 500 °C) typically have more oxygen-containing functional groups that facilitate ligand exchange and electrostatic attraction with As(V) ions. On the other hand, biochars produced at high temperatures (between 600 and 800 °C) tend to have larger surface areas and aromatic carbon structures, favoring physical adsorption mechanisms but exhibiting decreased chemical reactivity [194,195].

Although pristine biochar demonstrates moderate to good efficiency in removing As(V), its ability to adsorb As(III) is typically poor due to its negatively charged surface and the limited active binding sites for neutral or anionic As(III) species. To overcome this limitation, recent studies have focused on surface modification techniques that improve the affinity and selectivity of biochar for both arsenic species [196]. It has been shown that metal impregnation works quite well, especially when iron, aluminum, magnesium, manganese, or zirconium is used. Iron-modified biochars have a substantial binding capacity due to the formation of stable complexes between arsenic and iron oxyhydroxides or oxides, enabling dual-function removal through co-precipitation and adsorption [197,198].

Other advanced modifications, including the incorporation of layered double hydroxides (LDHs), acid/base activation, and nanoparticle impregnation, enhance porosity, surface charge, and chemical functionality. The Zr-loaded biochar and Mg–Al LDH-coated biochar have demonstrated better performance in binding As(V) through ion exchange and inner-sphere complexation mechanisms [199,200]. These modified biochars not only enhance arsenic adsorption capacity but also improve stability under variable pH and redox conditions. These systems are relatively inexpensive and scalable, but it is important to carefully consider their long-term stability and desorption risk in changing environmental circumstances.

Despite its many advantages, there are still difficulties in using biochar for large-scale arsenic remediation. Variations in feedstock availability, pyrolysis conditions, and biochar consistency can lead to performance inconsistency. It is also necessary to handle issues related to desorption or leaching of bound arsenic under fluctuating environmental conditions, such as pH shifts or redox changes. Long-term stability and regeneration potential also remain crucial concerns for field applications [201]. However, the environmental and economic sustainability of biochar makes it an attractive option for incorporation into hybrid systems combining nanotechnology, microbial remediation, and phytoremediation.

Recent research has extended into the development of multifunctional biochar composites capable of addressing co-contamination scenarios involving arsenic along with other heavy metals or organic pollutants. The combination of biochar with microbial consortia is being investigated to enhance arsenic transformation and immobilization in complex wastewater matrices [197,202]. Optimizing pyrolysis and functionalization procedures will be crucial to maximizing the environmental performance of biochar. A comparison of the several forms of biochar used to remove arsenic from contaminated water sources is shown in Table 4. The biochar feedstock, pyrolysis temperature (°C), preparation techniques, and adsorption capabilities are among the important parameters considered. Interestingly, biochars with numerous functional groups, higher surface area, and well-designed pores tend to absorb arsenic more effectively.

## 7. Nanotechnology-Based Approaches for Arsenic Remediation

Nanotechnology has developed as a transformative tool in environmental remediation, offering a wide surface area, increased reactivity, and adjustable surface chemistry [221]. Nanomaterials are highly effective at eliminating pollutants such as arsenic from water and wastewater [222]. Nanotechnology-based methods, including the use of nano-zero-valent iron (nZVI), metal oxide nanoparticles, and magnetic nanocomposites, provide high surface area, fast kinetics, and greater adsorption capacity. These methods are especially effective at eliminating trace amounts of As(III) and As(V). The application of nanomaterials, particularly engineered nanoparticles (ENPs), in arsenic remediation has demonstrated remarkable potential because of their strong adsorption capacity, excellent selectivity, and rapid kinetics [223].

Among the most extensively studied nanomaterials for arsenic removal are iron-based nanoparticles, such as nanoscale zero-valent iron (nZVI), iron oxide nanoparticles (e.g., maghemite, magnetite, ferrihydrite), and their composites [222]. These materials are effective because of their large surface area and high affinity for arsenic species, especially As(V). nZVI can reduce As(V) to As(III) and then immobilize it via co-precipitation and surface complexation [224]. However, aggregation and oxidation of nZVI limit its long-term performance. To overcome this issue, surface stabilization using polymers, biochar, or silica coatings has been employed to increase dispersion and avoid passivation [224].

Magnetic nanoparticles (MNPs) are becoming more popular due to their easy separation after treatment. Functionalized MNPs, such as Fe_3_O_4_ coated with chitosan, humic acid, or graphene oxide, have demonstrated remarkable removal effectiveness for both As(III) and As(V), with adsorption capabilities often exceeding those of conventional adsorbents [225]. The improvement in photocatalysts shows that the oxidation of As(III) and photocatalyst-impregnated adsorbents is an efficient and low-cost treatment method for arsenic removal from water [226]. Investigations were conducted for the photocatalytic oxidation of arsenite and the simultaneous removal of the produced arsenate from aqueous solution. In this system, an adsorbent was developed using an adsorbent of iron oxide and TiO_2_ on municipal solid waste melted slag [227]. A study performed using photoelectrocatalytic oxidation of As(III) over hematite photoanodes showed that hematite photoanodes are able to catalyze the oxidation of As(III) under solar illumination and can remove up to 90% As(III) within 24 h [228]. These materials offer the added benefit of their magnetic recoverability, enabling reuse and reducing secondary pollution.

Carbon-based nanomaterials, including graphene oxide (GO), carbon nanotubes (CNTs), and activated carbon nanocomposites, also demonstrate promising activity in arsenic adsorption [229]. The original form of CNTs shows limited arsenic removal efficiency, but their performance improves significantly when functionalized with metal oxides or polymers. GO-based nanocomposites exhibit high surface functionality when modified with iron hydroxides for enhanced arsenic affinity [229,230].

Metal–organic frameworks (MOFs) and nanocomposite hydrogels are an important class of nanoparticles that combine variable pore size, high porosity, and functional diversity [231]. MOFs like Zr-based UiO-66 and Fe-based MIL-100 have been developed to exhibit high arsenic uptake capacities and selectivity [232]. Despite their potential, MOFs have limitations in cost effectiveness, scalability, and stability under aqueous conditions or real-world applications [232].

Green synthesis of nanoparticles utilizing bacteria, fungi, or plant extracts is also an emerging eco-friendly approach. Biosynthesized nanoparticles, particularly biogenic iron and silver nanoparticles, offer environmental friendliness and reduced toxicity compared to chemically synthesized counterparts [233,234,235].

Although the nanotechnology-based approaches are highly promising, concerns remain regarding their cost of large-scale implementation, potential toxicity, environmental safety, difficulties with regeneration, and regulatory concerns over the discharge of nanoparticles into ecosystems may prevent their widespread application and ecological fate [233]. Recent studies demonstrated that metallic nanoparticles, especially silver nanoparticles (AgNPs) and titanium dioxide (nano-TiO_2_), can be accumulated in aquatic food chains. AgNPs accumulation increased markedly at producer levels (e.g., algae) transferred through zooplankton to fish, resulting in accumulation in the intestine, liver tissues, carcass, and embryos, causing oxidative stress and reproductive toxicity [236]. In addition, AgNPs can induce oxidative stress in *Clarias gariepinus*, *Danio rerio*, and *Ruditapes philippinarum*, affect the embryonal development of Sphaerechinus granularis and Arbacia lixula, cause behavioral changes and developmental defects in Paracentrotus lividus, and also accumulate in the intestines, gills, liver, and other organs of fish [237]. Terrestrial and plant systems are also affected by nanoparticles. They can enter the plant system through several pathways, such as root hairs, cracks on the leaf surface, and stomata, and consequently move through the plant system by diffusion [238]. Therefore, any application of nanomaterials in remediation must be accompanied by thorough environmental risk assessments and precautionary frameworks. Future research must focus on enhancing regeneration, stability in complex matrices, and combining nanomaterials with other technologies like microbial or phytoremediation to develop integrated, sustainable arsenic remediation systems [233]. Table 5 provides a comparative summary of various nanotechnology-based approaches for arsenic removal, including their core compositions, functionalization strategies, target arsenic species, adsorption capacities, and key advantages. These nanomaterials demonstrate diverse mechanisms and efficiencies, offering promising options for effective arsenic remediation.

## 8. Integrated and Hybrid Technologies for Arsenic Remediation

Individual treatment technologies may have limitations in effectively removing arsenic under variable environmental conditions. Integrated and hybrid systems are the most promising solutions that combine microbial, chemical, and physical methods have emerged especially for complex, mixed contaminant scenarios. Therefore, integrated and hybrid systems are being developed to improve treatment efficiency, cost-effectiveness, and environmental sustainability [259]. These systems produce synergistic benefits by combining oxidation, adsorption, filtration, and biological transformation. For example, integrating microbes with nanomaterials acts as electron shuttles that facilitate redox transformations of arsenic mediated by bacteria. In wastewater systems, the nanomaterials create biofilm and develop a favorable microenvironment for microbial colonization [260]. Studies have shown that *Acidithiobacillus ferrooxidans* exhibits As(III) removal efficiency of 88.26% when in contact with iron oxide nanoparticles [261], and *Shewanella* sp. demonstrates the ability of using organic exudates to facilitate As and Fe precipitation [262]. These approaches often overcome the shortcomings of individual processes by combining the best features of several physical, chemical, and biological techniques [263].

One of the most widely used hybrid approaches is the combination of adsorption and membrane filtration. For example, coupling iron oxide or activated alumina filters with nanofiltration (NF) or ultrafiltration (UF) membranes greatly enhanced arsenic removal, even in the presence of competing ions [264,265]. The membrane acts as a barrier against particles and colloidal debris, while the adsorbent eliminates arsenic species through surface complexation. These systems provide high removal efficiency (>99%) for both As(III) and As(V), especially when pre-oxidation is applied to convert the more mobile As(III) to As(V) [266].

Biological–chemical hybrid systems, such as biofilters integrated with iron dosing, have demonstrated significant promise. In these systems, iron-oxidizing bacteria (IOB) promote the oxidation of Fe(II) to Fe(III), facilitating in situ formation of iron hydroxides that adsorb arsenic [267]. This combination enhances both arsenic removal and iron cycling. For example, iron enrichment and biological sand filters have demonstrated long-term steady performance with minimal chemical input [268].

Another innovative hybrid involves phytoremediation combined with biochar or nanomaterials, which provide complementary benefits for arsenic-contaminated soils and wastewater [269]. Here, arsenic-accumulating plants such as *Pteris vittata* or *Lemna minor* are cultivated in substrates enriched with magnetic nanoparticles or functionalized biochar. The biochar improves arsenic immobilization in the root zone, while nanoparticles provide further removal through adsorption, forming a synergistic rhizo-filter [117,270]. Also, biochar derived from agriculture-based residue contains abundant functional groups and minerals that adsorb arsenic. In addition, biochar helps in the interaction of beneficial microbial communities with plant roots, further promoting arsenic transformation and stabilization. Such hybrid systems not only remove arsenic, but also improve soil health, water retention, and plant yield, making them sustainable, low-cost, and suitable for field-scale applications [164].

Electrochemical–adsorption hybrids are also becoming more popular. Electrocoagulation paired with adsorbents such as activated carbon or iron flocs removes arsenic efficiently, with less energy requirements and less sludge production compared to conventional chemical coagulation [271]. In such systems, iron or aluminum electrodes generate adsorptive hydroxide flocs in situ, binding arsenic as it passes through the reactor [271,272].

Microbial remediation integrating with nanotechnology, hybrid technology shows promise. For example, the combination of nano-iron particles with arsenic-oxidizing bacteria speeds up arsenic detoxification by converting As(III) to As(V) and then adsorbing it onto iron surfaces [273]. Such systems provide both redox transformation and potent adsorption, making them ideal for arsenic-containing groundwater treatment [266,274]. However, the hybrid systems are a straightforward approach since they can be customized to site-specific arsenic contamination circumstances. Furthermore, integrating hybrid technologies with automation, renewable energy, and real-time monitoring can significantly increase their sustainability and uptake in dispersed and rural areas [275]. However, hybrid systems often require higher operational complexity and upfront expenditures.

## 9. Comparative Analysis and Performance Evaluation of Arsenic Remediation Technologies

The selection of a suitable arsenic remediation strategy depends on multiple factors, including the oxidation state of arsenic, contaminant concentration, water chemistry, operating cost, environmental impact, and scale of application. Comparative studies of different technologies help to illuminate their relative strengths, limitations, and suitability across different environmental and socio-economic contexts. In terms of performance, technologies like membrane filtration and electrocoagulation provide high arsenic removal efficiency (>95%), but these are often energy-intensive and generate chemical sludge.

Therefore, a site-specific integration of several methodologies may be part of a balanced remediation plan, combining the accuracy and efficiency of nanotechnology or filtration techniques with the sustainability and cost-effectiveness of biological and bio-based solutions. Furthermore, scaling up laboratory-scale processes to industrial production while ensuring consistent nanoparticle distribution and stable properties remains a major challenge [276]. Ultimately, the choice must align with available infrastructure, the water quality objectives, and the local contamination profile. While many arsenic remediation technologies have been developed, their implementation status varies widely, ranging from industrial-scale applications to laboratory-scale. Well-established technologies such as membrane filtration, adsorption using activated alumina or iron-based media, and scorodite formation are commercialized. On the other hand, several emerging technologies such as nanomaterial–microbe hybrid systems, phytoremediation coupled with biochar, and engineered microbial consortia remain at the research and development stage. Table 6 demonstrates the comparative summary performances of arsenic remediation using different technologies.

## 10. Conclusions and Future Directions

Conventional chemical and physical methods such as coagulation, adsorption, and membrane filtration have demonstrated high As(V) removal efficiencies. However, these techniques often generate secondary waste, are energy-intensive, or require continuous input of chemicals. Emerging biological techniques, such as phytoremediation and microbial remediation, provide sustainable and cost-effective alternatives by utilizing the natural metabolic and accumulation capabilities of living organisms. Despite their potential, these approaches still face challenges in speed, scalability, and environmental management.

Biochar and modified biochar materials, made from agricultural residues and functionalized with metal oxides, have demonstrated excellent potential for arsenic immobilization, especially when integrated into soil or water filtration systems. Their low cost and local availability make them particularly attractive for large-scale deployment. On the other hand, nanotechnology-based solutions, such as metal oxide nanoparticles and magnetic nanocomposites, provide accuracy and high adsorption capacity for arsenic remediation. However, there are still issues with their cost, ecotoxicological effects, and safe disposal.

Several future research directions are required for the successful remediation of arsenic. The future of arsenic remediation lies in developing integrated, site-specific, and environmentally conscious strategies that combine the efficiency of engineered materials with the sustainability of biological systems. Hybrid technologies that combine microbial transformation with nanomaterials or plant-based systems with engineered biochar provide promise for addressing complex contamination scenarios. Pilot-scale studies and field validation are essential for evaluating the practical performance, cost, and adaptability of new materials and technologies. It is essential to do mechanistic research on arsenic speciation, binding kinetics, and long-term stability in diverse environmental matrices. Life-cycle assessments should be carried out to evaluate environmental footprints, energy demands, and waste generation associated with each technology. Education, community involvement, and policy support are also essential for successful implementation, particularly in rural and underserved regions where arsenic contamination is most common.

In conclusion, no single technology can be considered universally superior; instead, a multidisciplinary, flexible, and sustainable approach is key to overcoming the multifaceted challenge of arsenic contamination. Safe and arsenic-free environments will depend on continued innovation, supported by collaborative research, informed policymaking, and community involvement.

## Figures and Tables

**Table 1:** Microorganisms involved in arsenic remediation and their mechanisms.

Microorganism	Arsenic Resistance	Mechanism/Reaction	Arsenic Species Targeted	Adsorption/Removal% Efficiency	Optimal Conditions	Reference
*Exiguobacterium* *Profundum* PT2	Resists 25·2 mg/g As	Biosorption	As(V) and As(III)	Reduced 3·73 mmol in 48 h	pH 7; 37 °C	[65]
*Brevibacillus* sp.	Resists 265 mM of As(V) and 17 mM of As(III)	Reduction and oxidation	As(V) and As(III)	Removed ~40% of As	pH 7.1; 37 °C	[66]
*Bacillus aryabhatti*	Able to grow up to 500 mM As(V)	Reduction	As(V) and As(III)		pH 7; 60 °C	[67]
*Roseomonas* sp.	Resists 50 mM of As(V) and 2 mM of As(III)	Oxidation	As(V) and As(III)	Oxidized 2 mM As(III) in 60 h	pH 7.1; 37 °C	[68]
*Microbacterium*, *Micrococcus*, *Shinella*, and *Bacillus* sp.	Resists more than 400 mM As(V) and 8 mM As(III)	Reduction and oxidation	As(V) and As(III)	NA	pH 9; 30 °C	[69]
*Nocardioides* sp.	Resists 100 mM of As(V) and 5 mM of As(III)	Reduction	As(V) and As(III)	Reduced 2 mM As(V) in 36 h	pH 7; 37 °C	[68]
*Bacillus flexus* and *Acinetobacter junii*	Capable of growing at 150 mmol L^−1^ As (V) and 70 mmol L^−1^ As (III).	Biosorption	As(V) and As(III)	8 mg/g	pH 8; 30 °C	[70]
*Pseudomonas* sp. AK1	Able to grow at 13 mmol As (III).	Oxidation	As(III)	25% reduction in 72 h	pH 7; 30 °C	[71]
*Bacillus* sp., *Acinetobacter* sp.	Able to grow up to 300 and 350 mM As(V)	Bioaccumulation	As(V) or As(III)	848.33 mg/g dry cell weight	pH 7; 30 °C	[72]
*Pseudomonas* sp. AK9	Able to grow at 15 mmol As (III).	Oxidation	As(III)	25% reduction in 72 h	pH 7; 30 °C	[71]
*Acinetobacter* sp. and *Exiguobacterium* sp.	viable even at concentrations of 350 mM As(V) and up to As(III) 15 mM	Bioaccumulation	As(V) or As(III)	60%	pH 7; 35 °C	[73]
*Bacillus firmus*	tolerated 3 M As(V) and 75 mM As(III)	Oxidation	As(V) or As(III)	77% in 15 days	pH 9; 30 °C	[74]
*Bacillus* sp.	Viable at concentrations of 1000 mM As(V) and up to 70 mM of As(III)	Oxidation	As(III)	88%	pH 7; 33.5 °C	[75]
Brevibacterium sp. CS2	MIC of 280 mM As(V) and 40 mM of As(III)	Oxidation	As(III)	32 and 46% in wastewater and distilled water, respectively, in 8 days	pH 7; 37 °C	[76]
*Providencia rettgeri*	Viable at concentrations of 133.3 mM As(V)	Bioaccumulation	As(V)	NA	pH 7; room temp	[77]
*Lysinibacillus* sp.	MIC of 500 mM As(V)	Reduction	As(V)	Reduced 50%	pH 7; 37 °C	[4]
*Pseudomonas* sp.	Tolerable concentration of As(III) up to 3250 mg/L and As(V) up to 20,280 mg/L	Oxidation and reduction	As(V) or As(III)	Bacterium exhibited 48% of As(III) and 78% of As(V) transformation	pH 7.5; 25 °C	[78]
*Pseudomonas* sp. *HN-2*		Oxidation	As(III)	Oxidized 92.0% of As(III) to As(V) in 3 h	pH 7; 37 °C	[38]
*Pseudomonas* sp. and *Acinetobacter* sp.	MIC of 125 mM As(V) and 50 mM of As(III)	Oxidation and reduction	As(V) or As(III)	NA	pH 7; 37 °C	[79]
*Leclercia adecarboxylata*	Tolerated up to 100 mM As(V) and 10 mM As(III)	Reduction	As(V)	Harbored a typical As(V) reductase gene (arsC)	pH 7; 30 °C	[80]
*Pseudomonas aeruginosa*	The MIC was 7 g/L for As(V) and 1.4 g/L for As(III)	Oxidation and reduction	As(III)	98 mg/g	pH 7; 37 °C	[81]
*Bacillus cereus*	Resistant to 3000 mg/L of As	Oxidation reduction	As(V) or As(III)	Reduced 71.88% of As(III) and 85.72% of As(V)	pH 6.8; 30 °C	[82]
*Lysinibacillus boronitolerans*	Resistant to 3000 mg/L of As	Oxidation reduction	As(V) or As(III)	Reduced 71.88% of As(III) and 85.72% of As(V)	pH 6.8; 30 °C	[82]
*Bacillus* sp.	MIC of 500 mM of As(V)	Reduction	As(V)	As(V) reduction efficiency was optimized to 72%	pH 6.8; 30 °C	[83]
*Micrococcus* sp.	Capable of growing at 150 mmol L^−1^ As (V) and 70 mmol L^−1^ As (III).	Oxidation	As(V) or As(III)	Reduced 91.04%	pH 7; 30 °C	[84]
*Bacillus* sp.	MIC As(V) up to 4500 ppm and 600 ppm of As(III)	Oxidation and reduction	As(V) or As(III)	51.45% As(III) and 53.29% As(V)	30 ± 1 °C	[85]
*Aneurinibacillus aneurinilyticus*	MIC As(V) up to 4500 ppm and 600 ppm of As(III)	Oxidation and reduction	As(V) or As(III)	51.99% As(III) and 50.37% As(V)	30 ± 1 °C	[85]

NA—not applicable.

**Table 2:** Comparison of different bio-based adsorbents for arsenic removal.

Adsorbent/Material	Modification/Type	As Species Targeted	Adsorption Capacity (mg/g)/Removal Efficiency (%)	Optimal pH	Mechanism/Key Advantages	Reference
Orange peel	Modified titanium dioxide (TiO_2_)		10.91 mg/g	4.2	High surface area, eco-friendly	[121]
Shrimp-based chitosan	Modified by 1.5% HCl and 5% NaOH	As(V)	98.5%; 15.92 mg/g	7	Abundant supply at low costs	[20]
Lemon peel	NB	As(III)	72.34%	6	Low-cost and sustainable biosorbent	[122]
Green tea Waste	Modified by Ca(OH)_2_	As(III)	0.4212 mg/g	3; 33 °C	High surface area, eco-friendly	[123]
Rice husk	Alkaline activation	As(V)	15–30 mg/L	3	Agro-waste utilization	[124]
Rice husk	NB	As(V)	90.7%	8	Cost-effective and biodegradable	[125]
*Citrus limetta* peel	Zirconium-modified	As(V)	75.86 mg/g	5.8	Low-cost and sustainable biosorbent	[126]
Pomegranate peels	Modified by TiO_2_	As(III)	76.92 mg/g	pH = 7, T = 25 °C	Cheap, easy-going	[127]
Orange peel	Ca(OH)_2_-modified	As(V)	43.69 mg/g	5.5	Low-cost and eco-friendly adsorbents	[128]
Watermelon rind	Modified by citric acid	As(III), As(V)	As(III) (99%) and As(V) (98%)	8.2	Cheap, easy-going	[129]
Banana peel	Calcium nitrate, diammonium Hydrogen phosphate, sulfuric acid, ferric nitrate -modified	As(V)	98.7%	4–6	Ligand exchange, electrostatic	[130]
Sugarcane bagasse	Thiol-functionalized	As(III), As(V)	28.57 mg/g	7	Low cost, green	[131]
Mango peel	Zr(IV)	As(III)	87.32%; 45.52 mg/g	10.18	Adsorption via carbon matrix	[132]
Sugarcane bagasse	Activation using H_3_PO_4_	As(III)	6.69 mg/g	8	Abundant supply at low costs	[133]
Bamboo charcoal	Iron-modified	As(III), As(V)	7.23 mg/g	4–5	High surface area, large pore volume, and low cost	[134]
Pomegranate waste	Fe(III)-loaded	As(III)	50 mg/g	9	Low-cost bioadsorbent	[135]
Sawdust	Treated using ZrO_2_	As(III), As(V)	29 mg/g (AsIII) and 12 mg/g (AsV)	7	Environmentally friendly and cost-effective	[136]
Apple peel	Zirconium-loaded	As(III), As(V)	5.68 mg/g	2–6	Low cost, green	[137]
Watermelon peel	NM	As(III), As(V)	99.99%	5.5–7.5	Low cost, high efficiency	[138]
Java plum seeds	NM	As(III), As(V)	78% As(III) and 67% As(V)	7 for As(III) and 5.3 for As(V)	Inexpensive, effective, and sustainable	[139]
Egg shell	NM	As(III), As(V)	87% As(III) and 71% As(V)	7 for As(III) and 4 for As(V)	Inexpensive, effective, and sustainable	[139]
Water chestnut shell	NM	As(III), As(V)	75%	7	Inexpensive, effective, and sustainable	[139]
Corn cob	NM	As(III), As(V)	67%	7	Inexpensive, effective, and sustainable	[139]
Tea waste	NM	As(III), As(V)	74%	7	Inexpensive, effective, and sustainable	[139]
Pomegranate peel	NM	As(III), As(V)	65%	9	Inexpensive, effective, and sustainable	[139]
Rice polish	NM	As(III), As(V)	As(III) (41.18 μg/g) and As(V) (49 μg/g)	6.84 for As(III) and 4.29 for As(V)	Cheap, easy-going	[140]
Chir pine leaves	NM	As(III), As(V)	3.27 mg/g	4	Exothermic, spontaneous, and favorable	[141]
Blue pine wood shavings	NM	As(III), As(V)	97%	10	Simplicity and easy operation, and handling	[142]
Walnut shell	NM	As(III), As(V)	88%	10–11	Simplicity and easy operation, and handling	[142]
Chick pea testa	NM	As(III), As(V)	35%	8	Simplicity and easy operation, and handling	[142]
Rice husk	NM	As(III), As(V)	96%	6.5	Environmentally friendly, cost-effective, and biodegradable	[143]

NM—not modified.

**Table 3:** Comparison of common plants used in arsenic phytoremediation of arsenic.

Plant Species	Type	Max As Uptake (mg/kg)	Mechanism	Advantages	Reference
*Pteris vittata*	Fern	>2000	Phytoextraction	Fast-growing, high As accumulation	[151]
*Pteris vittata*	Fern	1860	Phytoextraction and phytostabilization	Potential candidate for As removal in soils and sediments	[166]
*Pteris vittata* L.	Fern	7215–11,110	Phytoaccumulation	Capable of co-hyperaccumulating high As levels	[167]
*Artemisia divarica*	Dicotyledons	47.26	Phytoextraction	Low-cost, prevents pollution, enables fast recycling	[168]
*Pteris ensiformis*	Fern	1091	phytoextraction	high biomass, wide occurrence, and rapid growth	[169]
*Pteridium aquilinum*	Fern	622	Rhizofiltration	Eco-friendly, solar-powered	[170]
*Pteris cretica*	Fern	4875	Phytoaccumulation	Phosphomolybdic acid from Pteris cretica is converted to Mg_3_(PO_4_)_2_, a potential fertilizer	[171]
*Eruca sativa*	Herb	0.1560–0.1630	Phytoaccumulation	Fast growth, high biomass	[172]
*Azolla caroliniana*	Fern	386.1	Phytoaccumulation	Rapid growth and reproduction, high surface area	[173]
*Ceratophyllum* *demersum*	Hornwort	60%	Phytoaccumulation	Fast-growing, high As uptake, low maintenance.	[174]
*Cladophora* sp.	Algae	6 mg/ L	Phytoaccumulation	Rapid growth, high surface area	[175]
*Chlorodesmis*	Algae	4 mg/L (40–50%)	Phytoaccumulation	Fast growth rate, low-cost, and eco-friendly	[175]
*Arundo donax*	Reed	600 μg/ L	Phytoaccumulation	Plant growth was observed within an As concentration range of 50–600 μg/L	[176]
*Lemna* *minor*	Duckweed	8.70–15.02%	Rhizofiltration	Low maintenance and cost-effective	[177]
*Eichornia crassipes*	Water hyacinth	39.2%	Rhizofiltration	Extensive root system and cost-effective	[107]
*Phragmites* *karka*	Tall reed	46%	Phytoaccumulation	Large biomass and surface area	[178]
*Pteris vitatta*	Fern	3.5–11.4%	Phytoaccumulation	Fast-growing, large biomass	[179]
*Genetically Modified Arabidopsis thaliana*	Herb	As uptake 28 µg/g in the shoot and 2400 µg/g in the root	Phytoaccumulation	Small size, low space requirement, and easy cultivation	[14]
*Colocasia esculenta*	Angiosperm	89%	Phytoaccumulation	High biomass, efficient uptake, and accumulation	[180]
*Lemna valdiviana*	Duckweed	1190 mg/kg (82% removal)	Phytoextraction	Low maintenance and cost-effective	[181]
*Wolfia globosa*	Watermeal	>1000 mg/kg	Phytoaccumulation/ Phytofiltration	High surface area, fast growth rate	[182]
*Vallisneria natans*	Grass	58.11–66.21%	Phytoextraction	Low maintenance and fast growth rate	[183]
*Eichhornia crassipes*	Water hyacinth	83%	Phytoaccumulation/ Phytoextraction	Extensive root system and cost-effective	[184]
*Pistia stratiotes*	Water lettuce	Root and leaf content 1120.40 µg/g and 31.60 µg/g, respectively	Phytoaccumulation	Free-floating, rapid growth, and high biomass	[185]
*Lemna minor*	Duckweed	>70%	Phytoaccumulation	Low maintenance and cost-effective	[186]
*Viola macedonica*	Herb	783 mg/kg	Phytostabilization	Extensive root system, adaptability to various soils	[187]
*Viola arsenica*	Herb	2124 mg/kg	Phytostabilization	Extensive root system, adaptability to various soils	[187]
*Leucaena leucocephala*	Dicotyledons	6.83	Bioaccumulation and phytoextraction	Potential for both fertility improvement and heavy metal(loid) hazard prevention	[188]
*Acacia mangium*	Dicotyledons	1549	Phytostabilization	Able to survive on arsenic- conc. up to 500 mg/kg	[189]
*Retama sphaerocarpa*	Shrub	>88%	Phytostabilization	Deep root system, biomass production, and drought tolerance	[187]

**Table 4:** Comparative summary of different biochar types used for arsenic removal.

Biochar Type	Pyrolysis Temp (°C)	Modification	Arsenic Species Targeted	Removal%/Adsorption Capacity (mg/g)	Reference
Corncob biochar	450	Amendment materials with Fe, Mn	As(III), As(V)	Reduced 51.2–54.1%	[203]
Coffee husk and corncob biochar	600	Impregnated with ZnO	As(V)	25.9 mg/g	[204]
Pomegranate peels	400	Impregnated with TiO_2_	As (III)	76.92 mg/g	[127]
Perilla leaf biochar	700	Not modified	As(III), As(V)	(97–100%)	[205]
Oak wood biochar	500	Not modified	As(III), As(V)	4 mg/g (92 to 100%)	[206]
Chinese traditional medicine dregs waste biochar	450	Iron-doped TiO_2_ Modification	As(III)	58.45 mg/g	[207]
Henequen fibers biochar	260	FeCl_3_·6H_2_O modification	As(V)	8.98 mg/g	[208]
Brown seaweed biochar	400	FeCl_3_·6H_2_O modification	As(V)	0.83 mg/g 96.7%	[209]
Rice straw biochar	500	FeSO_4_·7H_2_O and FeCl_3_·6H_2_O	As(V)	26.9 mg/g	[210]
Bamboo biochar	700	Fe_3_O_4_ modified	As(V)	13.9 mg/g (100%)	[211]
Wood waste biochar	600	Promoted by FeCl_3_ and KMnO_4_	As (III)	81% and 0.72 mg/g	[212]
Corn straw biochar	600	Treated with FeCl_3_	As(V)	6.80 mg/g	[213]
Rice straw biochar	500	Using FeCl_3_ modification	As(V)	28.49 mg/g	[214]
Cotton stalks biochar	400	Treated with nitric acid (HNO_3_) and Base (NaOH)	As(III)	157 µg/g	[215]
Durian shells biochar	500	Fe-ZrO-modified	As(III), As(V)	As(III) 46.7 and As(V) 47.8 mg/g	[216]
Pine wood biochar	600	Birnessite	As(V)	910 µg/g	[217]
Pine wood biochar	600	Mn oxide	As(V)	590 µg/g	[217]
Corncob biochar	350	Modified with zirconium (CCB@Fe_3_O_4_-Zr with Zr to Fe_3_O_4_)	As(III), As(V)	81% (As(III)), 99% (As(V)) removal	[200]
Chitosan biochar	531.96	Modified sodium alginate (Zr-CTS/SA)	As(III), As(V)	43.19 mg/g (As(III)), 76.78 mg/g (As(V))	[218]
Pristine biochar	300	Iron (Fe) and binary zirconium–iron (Zr–Fe)-modified	As(V)	67.28	[219]
Fruit waste biochar	500	Not modified	As(V)	88.8 ± 0.04%	[220]

**Table 5:** Comparative summary of nanotechnology-based approaches for arsenic removal.

Nanomaterial	Functionalization/Support	Target Arsenic Species	Adsorption Capacity (mg/g)	Advantages	Reference
Zero-valent iron (Z-NZVI)	Zeolite	As(III)	11.52 (mg/g)	Improved kinetics, high surface area, and dispersion	[239]
CNT/Ch, and PDA@CNT/Ch	Chitosan aerogel	As(III)	94%	Highly functional at low pH	[240]
SiO_2_@Fe_3_O_4_@MBT	Silica (SiO_2_), Fe_3_O_4_ is embedded/coated for magnetism	As(V)	95.77%	Fast adsorption and high surface area	[241]
Cellulose nanofibril aerogels/Fe-IONPs	Cellulose nanofibril aerogels	As(III), As(V)	As(III) 48 mg/g, As(V)91 mg/g	High porosity	[242]
Graphene oxide/CuFe_2_O_4_ foam	Graphene oxide (GO) and copper ferrite composite	As(III), As(V)	44 (mg/g)	High adsorption capacity, eco-friendly, and reusable	[243]
Magnetic Fe_3_O_4_@CuO nanocomposite	Graphene oxide (GO)	As(III), As(V)	As(III) 70.36 mg/g; As(V) 62.60 mg/g	High adsorption capacity, eco-friendly, and durable	[244]
Core/shell structural nZVI/Mn oxide	Manganese oxide shell	As(III), As(V)	29.4	Improved selectivity, high adsorption capacity	[245]
Fe_3_O_4_ NP-NCNT hybrid	Agglomeration-free Fe_3_O_4_	As(III), As(V)	69% As(III), 35% As(V)	High affinity for arsenic, regeneration	[246]
Hydrophilic poly(vinyl alcohol) (PVA) nanofibers	Polymeric matrix	As(V)	3.5 mg/g	Excellent dispersion and surface availability of ions	[247]
Iron-based metal–organic frameworks MIL-101 [NH2-MIL-101(Fe)]	Iron-based metal	As(III), As(V)	As(V) and As (III) were 148 and 153 mg/g	Fast kinetics, high surface area and porosity	[248]
Fe_3_O_4_@Nzvi-PEI	Polyethyleneimine	As(III), As(V)	95.8% As(III), 90.5 As(V)	Fast kinetics, high reduction capacity	[249]
Zr-UiO-66-SH-A	Zirconium oxide (ZrO_2_)	As(III), As(V)	As(III) and As(V) 90.7 and 98.8 mg/g	High affinity for arsenic, regeneration, and reusability	[250]
AC/Fe_3_O_4_	Magnetic iron oxide	As(III), As(V)	As(III) 70% and As(V) 29%	High adsorption capacity, high specific surface area.	[251]
Cerium oxide (CeO_2_) nanoparticles (NPs)	Cerium oxide	As(III), As(V)	451 mg/g As(III), 119 mg/g As(V)	Eco-friendly, large surface area, and porosity	[252]
Nano-alumina-coated carbon microspheres (Al-CMs)	Carbon microsphere (CM) core	As(V)	68 mg/g	Reusability of adsorbent	[253]
Alginate-coated superparamagnetic Iron Oxide nanoparticles (SPIONs)	Alginate beads (SPIONs-Alg)	As(V)	99% (240.08 mg/g)	High adsorption capacity, eco-friendly, and biocompatible	[254]
Copper-doped ZIF-8 nanomaterials	Zeolitic imidazolate	As(III), As(V)	238.11 mg/g As (III) and 10–350 mg/g As(V)	Fast kinetics, reusability, and high surface area	[255]
Zinc oxysulfide nanomaterials (ZnOxS1-x)	Silica (SiO_2_)	As(III), As(V)	299.4 (99.9%)	High affinity for arsenic, low cost, and abundance	[256]
Starch-functionalized maghemite nanoparticles (g-Fe_2_O_3_@starch)	Starch polymer	As(III)	8.88	Cost-effective synthesis, eco-friendly and biocompatible	[257]
Zr-metal–organic framework (UiO-66)-derived t-ZrO_2_	Zirconium oxide (ZrO_2_)	As(III), As(V)	352.1 mg/g	Faster adsorption rate and ultrahigh uptake	[258]

**Table 6:** Comparative performance summary of arsenic remediation technologies.

Technology	Mechanism	Efficiency	Cost	Advantages	Limitations	Suitable Application
Microbial Remediation	Oxidation/reduction	Moderate–High	Low–Medium	Eco-friendly, selective transformation	Slower process, sensitive to environmental conditions	Groundwater, wastewater, wetlands
Phytoremediation	Uptake and accumulation by plants	Moderate	Low	Low-cost, green, improves soil health	Time-consuming and biomass disposal issues	Contaminated soil and wetlands
Biochar and Modified Biochar	Adsorption and immobilization	Moderate–High	Low–Medium	Abundant materials, easy application	Risk of desorption depends on biochar quality	Soil remediation, filtration units
Nanotechnology-Based Methods	Adsorption, redox transformation	High (>95%)	Medium–High	High capacity, fast kinetics	Potential nanotoxicity, high cost	Point-of-use water treatment
Membrane Filtration	Size exclusion/adsorptive filtration	Very High (>99%)	High	Precise removal, effective even at low concentrations	Expensive, fouling, energy-demanding	Urban and industrial wastewater
Adsorption	Adsorption and immobilization	High	Low-medium	Ease of operation, low cost, fast kinetics	Requires solid waste disposal, Non-destructive for contaminants	Groundwater, urban and industrial wastewater
Electrocoagulation	In situ coagulant generation	High	Medium	Sludge-free, chemical-less	Power needs, electrode passivation	Decentralized treatment plants
Hybrid/Integrated Systems	Combined bio-physico-chemical	Very High	Variable	Synergistic effects, site-specific design	Requires monitoring and integration expertise	Groundwater, urban and industrial wastewater

Note: Cost estimation is presented in relative terms (low, medium, high) as reported across multiple studies, since absolute cost values vary significantly depending on scale, location, and operational conditions.

## Data Availability

All the data used in this study are presented in the manuscript.
